# The predictive value of masticatory function for adverse health outcomes in older adults: a systematic review

**DOI:** 10.1016/j.jnha.2024.100210

**Published:** 2024-03-14

**Authors:** Menke J. de Smit, Willemke Nijholt, Mieke H. Bakker, Anita Visser

**Affiliations:** a Department of Gerodontology, Center for Dentistry and Oral Hygiene, University of Groningen, University Medical Center Groningen, Groningen, The Netherlands; b Department of Gerodontology, College of Dental Sciences, Radboud University Nijmegen Medical Centre, Nijmegen, The Netherlands

**Keywords:** Masticatory function, Frailty, Sarcopenia, Malnutrition

## Abstract

Masticatory function is associated with a variety of health outcomes. The aim of this systematic review is to clarify the predictive value of masticatory function for adverse health outcomes, such as frailty, sarcopenia and malnutrition, in older adults.

An online literature search covered articles published in English or Dutch in three databases (PubMed, Embase and CINAHL, last searched November 4th 2022). Inclusion criteria were: an observational study design, focus on adults aged ≥65 years and evaluation of the association between masticatory function and health outcomes. Reviews and articles published before the year 2000 were excluded. Methodological quality and risk of bias were assessed using the Quality Assessment Tool for Observational Cohort and Cross-Sectional Studies of the U.S. National Institutes of Health. Study population demographics, methods for assessing masticatory function, and the association between masticatory function and adverse health outcomes were extracted.

From the 34 included studies, 5 studies had a prospective design, 2 had a retrospective design, and the other 27 studies had cross-sectional design. The majority of the studies were conducted in Japan (74%, n = 26). Twenty studies (59%) used one indicator for masticatory function, the other 41% used two (n = 9) or more (n = 5) indicators. Masticatory function was most frequently assessed with the maximum occlusal force (MOF) (79%, n = 27). The identified health outcomes were clustered into 6 categories: physical parameters and sarcopenia, history of falling, nutritional status, frailty, cognitive function and mortality.

Despite the complex and multidimensional character of both masticatory function and most identified adverse health outcomes, some significant associations were reported. Prospective studies showed that reduced masticatory function in older adults is associated with incidence of frailty and frailty progression, cognitive decline and all-cause mortality. Regarding the other identified adverse health outcomes, i.e., physical measures and sarcopenia, history of falling and nutritional status, only cross-sectional studies were available and results were less concordant. As all prospective studies showed that reduced masticatory function in older adults is associated with adverse health outcomes, prevention of decline of masticatory function by adequate oral care may contribute to healthy ageing.

## 1 Introduction

As frailty, sarcopenia and malnutrition are highly prevalent in older adults, it is important to identify these conditions at an early stage. To assist in identification of these clinical conditions, indicators (biological markers) of underlying health problems and propensity to diseases, conditions or impairments can be helpful [1]. An example of such a biological marker is handgrip strength. Handgrip strength is often used as an indicator of overall health status in older adults [2]. Handgrip strength is an important predictor of mobility disability and decline in activities of daily living [3,4]. Furthermore, handgrip strength can predict cognitive impairment in older adults [5]. Another potential biological marker for frailty and sarcopenia is masticatory function, as maintenance of a nutritionally complete diet is important in preventing these clinical conditions [6]. Related terms for masticatory function are masticatory ability, masticatory efficiency, masticatory performance, chewing function or chewing efficiency, and a variety of methods is reported to describe the masticatory process. However, these terms are used interchangeably and sometimes involve different methodologies. As standardization of terms is essential to allow for comparison among different studies, a proposal for consensus on the terminologies and methodologies for masticatory assessment was provided by Gonçalves et al. in 2021 [7], in which they describe subjective and objective assessments of masticatory function. Subjective assessment holds self- or proxy-assessed masticatory function by questionnaires and interviews. Objective assessment is divided into direct or indirect measures. Direct objective assessment includes comminution test, mixing ability test and other chewing tests in which the participant is asked to chew on test food such as peanuts, gummy jelly or two-colored chewing gum for a fixed number of cycles. Direct objective assessment includes the comminution test for swallowing threshold, in which the number of cycles, particle size, or textual properties until swallowing threshold is measured. Indirect objective assessment of masticatory function includes jaw muscle activity, bite force or maximum occlusal force (MOF) and tongue and lip function. The MOF represents the maximum effort exerted while biting on a recording device, expressed in Newton [8]. Jaw muscle activity is commonly recorded from the masseter and temporal muscles using electromyography. Although not mentioned in the masticatory assessment overview of Gonçalves et al. [7], quality and quantity of the masticatory muscles is also measured by imaging techniques such as ultrasound, computed tomography or magnetic resonance images.

A systematic understanding of the association of masticatory function and adverse health outcomes in older adults is lacking. This review aims to clarify the value of masticatory function as an early indicator for adverse health outcomes in older adults, such as frailty, sarcopenia and malnutrition.

## 2 Methods

The Preferred Reporting Items for Systematic Reviews and Meta-Analyses (PRISMA) 2020 statement was followed [9].

### 2.1 Search strategy

The electronic databases PubMed, Embase and the Cumulative Index to Nursing and Allied Health Literature (CINAHL) were searched for articles published in English or Dutch, all last searched November 4th 2022. Concept of interest was defined as masticatory function in relation to adverse health outcomes in older adults. Search terms related to these three research domains were selected: (1) Masticatory function: occlusal force, masticatory performance, masseter/temporal muscle thickness, (2) Health outcomes: activities of daily life, body mass index, cognitive function, frailty, hand grip strength, mortality, muscle strength, nutritional status), and (3) Older adults: adults, elderly, aging. The complete search strategy can be found in the supplementary materials (Appendix).

### 2.2 Health outcomes

Studies evaluating the association between masticatory function and (adverse) health outcomes were included in this systematic review. The following (adverse) health outcomes were evaluated: activities in daily life, body mass index, cognitive function, frailty, hand grip strength, mortality, muscle strength and nutritional status. All studies that specifically investigated the association between masticatory function and the abovementioned outcomes were considered eligible. All types of assessment methods for the health outcomes, for example, self-reported questionnaires, clinical observations, biochemical tests and anthropometric measurements were included in this systematic review. Specifically for frailty, the physical, social and psychological domain were considered eligible.

### 2.3 Selection procedure

Endnote software (Disc bv, endnote X9.3.3) was used to insert the search hits from the three databases and duplicates were removed. Articles were considered eligible if they met the following inclusion criteria: published from the year 2000 onwards, full text available in English or Dutch, an observational study design, focus on older adults aged ≥65 years and evaluation of the association between masticatory function and (adverse) health outcomes. Reviews and articles published before the year 2000 were excluded. The complete selection procedure was carried out by two reviewers, independently of each other. First, all titles and abstracts were screened and relevant articles were selected based on the inclusion criteria. Subsequently, full text articles were assessed. After that, the methodological quality and risk of bias of the articles was evaluated (see next section). The reviewers discussed their opinion to reach consensus on inclusion of the article after each phase (i.e., title/abstract, full text and methodological quality). In case of lack of consensus between the two reviewers, a third reviewer was consulted.

### 2.4 Methodological quality assessment and risk of bias

Methodological quality and risk of bias of the articles was assessed using the Quality Assessment Tool for Observational Cohort and Cross-Sectional Studies [10]. This instrument contains 14 items, with higher scores indicating better methodological quality and low risk of bias. If an item of the quality instrument was not applicable for the concerning study design or the information regarding that item was not reported, this item was scored with ‘NA’ or ‘NR’. For each item present, one point was awarded and added up to an overall score. The methodological quality of the studies was qualified as ‘good’ with a minimum of 10 points, ‘fair’ with a score of 8 or 9 points and ‘poor’ if 7 points or less were scored.

### 2.5 Data analyses

From the included studies, study population demographics, methods for assessing masticatory function, health outcomes, and the relation between masticatory function and health outcomes were extracted. The studies will be clustered into six categories: physical parameters and sarcopenia, history of falling, nutritional status, frailty, cognitive function and mortality. The studies will be stratified by study design and shown in evidence tables in which both study characteristics and the quality of the studies are shown.

## 3 Results

### 3.1 Selection procedure

**Fig. 1:**
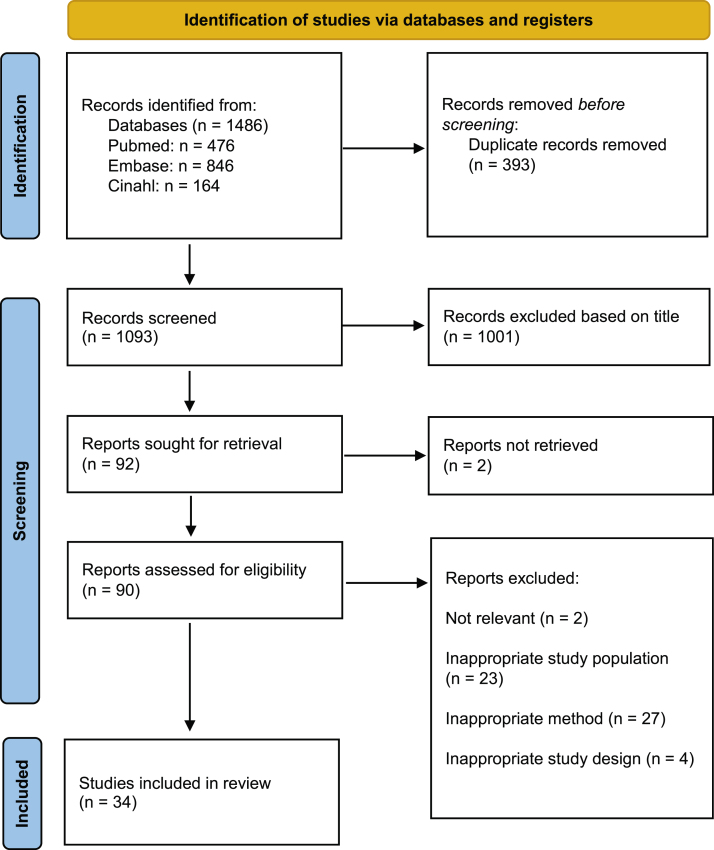
PRISMA 2020 flow diagram of the selection process [9].

An overview of the selection process is shown in Fig. 1. Initially, 1486 studies were identified. After screening on title, abstract and full text, 34 observational studies were included for analysis.

### 3.2 Study design

Five studies had a prospective and/or longitudinal design [[11], [12], [13], [14], [15]], 2 studies had a retrospective design [16,17], the remaining 27 (79%) were cross-sectional studies.

### 3.3 Methodological quality assessment and risk of bias

Total quality scores and risk of bias in the studies are presented in Table 1. The quality score ranged from 8 [[18], [19], [20], [21], [22], [23], [24]] to 13 [16]. Eleven studies (32%) were qualified as good and 23 studies (68%) as fair. None of the included studies were qualified as poor.

**Table 1:** Methodological quality and risk of bias of the included articles according the Quality Assessment Tool for Observational Cohort and Cross-Sectional Studies [10].

	1. Clear research question?	2. Study population specified?	3. Participation rate >50%	4. Selection of study population described?	5. Power analysis?	6. Exposure prior to outcome?	7. Sufficient timeframe?	8. Different levels outcome?	9. Valid exposure?	10. Exposure over time?	11. Reliable and valid outcome?	12. Blinding	13. Loss to follow up?	14. Adjusted to confounding?	Total
Quality scoring good (≥10 points)															
Gonzalez- Fernandez et al. (2021) [27]	Yes	Yes	Yes	Yes	No	Yes	NA	Yes	Yes	NA	Yes	Yes	NA	Yes	10 3xNA
Hatta et al. (2020) [15]	Yes	Yes	Yes	Yes	No	Yes	Yes	Yes	Yes	Yes	Yes	NR	No	Yes	11 1 x NR
Iinuma et al. (2016) [11]	Yes	Yes	Yes	Yes	No	Yes	Yes	Yes	Yes	Yes	Yes	NR	Yes	Yes	11
Ikebe et al. (2018) [33]	Yes	Yes	Yes	Yes	yes	Yes	NA	Yes	Yes	NA	Yes	NR	NA	Yes	10 1x NR 1x NA
Iwasaki et al. (2020) [40]	Yes	Yes	Yes	Yes	No	Yes	Yes	Yes	Yes	Yes	Yes	NR	No	Yes	11 1x NR
Ohi et al. (2020) [13]	Yes	Yes	Yes	Yes	No	Yes	Yes	Yes	Yes	Yes	Yes	NR	Yes	Yes	12 1x NR
Takeshita et al. (2016) [32]	Yes	Yes	Yes	Yes	Yes	Yes	NA	Yes	Yes	NA	Yes	NR	NA	Yes	10 3xNA 1x NR
Waduud et al. (2020) [16]	Yes	Yes	Yes	Yes	Yes	Yes	Yes	Yes	Yes	Yes	Yes	NR	Yes	Yes	13 1x NR
Wallace et al. (2017) [17]	Yes	Yes	NR	Yes	No	Yes	Yes	Yes	Yes	Yes	Yes	NR	Yes	Yes	12 2xNR
Yamaguchi et al. (2019) [39]	Yes	Yes	Yes	Yes	Yes	Yes	NA	Yes	Yes	NA	Yes	NR	NA	Yes	10 3xNA 1xNR
Yoshida et al. (2022) [43]	Yes	Yes	Yes	Yes	Yes	Yes	NA	Yes	Yes	NA	Yes	Yes	NA	Yes	11 3xNA
Quality scoring fair (8 or 9 points)															
Aquilanti et al. (2020) [25]	Yes	Yes	Yes	Yes	No	Yes	NA	Yes	Yes	NA	Yes	Yes	NA	No	9 3x NA
Aquilanti (2021) [24]	Yes	Yes	Yes	Yes	No	Yes	NA	Yes	Yes	NA	Yes	NR	NA	No	8 3x NA 1x NR
Eto et al. (2018) [41]	Yes	Yes	Yes	Yes	No	Yes	NA	Yes	Yes	NA	Yes	Yes	NA	No	9 3x NA
Hasegawa et al. (2019) [44]	Yes	Yes	Yes	Yes	No	Yes	NA	Yes	Yes	NA	Yes	Yes	NA	No	9 1 x NR 3 x NA
Hasegawa et al. (2019) [29]	Yes	Yes	Yes	Yes	No	Yes	NA	Yes	Yes	NA	Yes	NR	NA	Yes	9 1x NR 3x NA
Hihara et al. (2020) [19]	Yes	Yes	Yes	Yes	No	Yes	NA	Yes	Yes	NA	Yes	NR	NA	No	8 1 x NR 3 x NA
Hirao et al. (2014) [20]	Yes	Yes	Yes	Yes	No	Yes	NA	Yes	Yes	NA	Yes	NR	NA	No	8 1 x NR 3 x NA
Horibe et al. (2018) [12]	Yes	Yes	No	Yes	No	Yes	Yes	Yes	Yes	Yes	Yes	NR	No	No	9 1x NR
Horibe et al. (2018) [21]	Yes	Yes	Yes	Yes	No	Yes	NA	Yes	Yes	NA	Yes	NR	NA	No	8 2x NA 1x NR
Iinuma et al. (2012) [42]	Yes	Yes	Yes	Yes	No	Yes	NA	Yes	Yes	NA	Yes	NR	NA	Yes	9 3xNA 1xNR
Iwasaki et al. (2018) [14]	Yes	Yes	Yes	Yes	No	Yes	NA	Yes	Yes	NA	Yes	NR	NA	Yes	9 3xNA 1x NR
Komiyama et al. (2020) [34]	Yes	Yes	No	Yes	No	Yes	NA	Yes	Yes	NA	Yes	NR	NA	No	9 3x NA 1x NR
Kugimiya et al. (2019) [36]	Yes	Yes	Yes	Yes	No	Yes	NA	Yes	Yes	NA	Yes	NR	NA	Yes	9 3x NA 1x NR
Laguna et al. (2015) [26]	Yes	Yes	Yes	Yes	No	Yes	NA	Yes	Yes	NA	Yes	NR	NA	No	9 2x NA 1x NR
Miura et al. (2003) [22]	Yes	Yes	Yes	Yes	No	Yes	NA	Yes	Yes	NA	Yes	NR	NA	No	8 3x NA 1x NR
Morita et al. (2019) [35]	Yes	Yes	Yes	Yes	No	Yes	NA	Yes	Yes	NA	Yes	NR	NA	Yes	9 3xNA 1x NR
Okada et al. (2010) [30]	Yes	Yes	Yes	Yes	No	Yes	NA	Yes	Yes	NA	Yes	NR	NA	Yes	9 3xNA 1x NR
Okada et al. (2015) [31]	Yes	Yes	Yes	Yes	No	Yes	NA	Yes	Yes	NA	Yes	NR	NA	Yes	9 3xNA 1x NR
Okamoto et al. (2019) [37]	Yes	Yes	Yes	Yes	No	Yes	NA	Yes	Yes	NA	Yes	NR	NA	Yes	9 2xNA 1x NR
Ozsürekci et al. (2022) [28]	Yes	Yes	Yes	Yes	No	Yes	NA	Yes	Yes	NA	Yes	NR	NA	Yes	9 3xNA 1xNR
Shimazaki et al. (2020) [23]	Yes	Yes	No	Yes	No	Yes	NA	Yes	Yes	NA	Yes	NR	NA	Yes	8 3xNA 1x NR
Watanabe et al. (2017) [38]	Yes	Yes	Yes	Yes	No	Yes	NA	Yes	Yes	NA	Yes	NR	NA	Yes	9 3xNA 1xNR
Weijenberg et al. (2015) [18]	Yes	Yes	No	Yes	No	Yes	NA	Yes	Yes	NA	Yes	NR	NA	Yes	8 3xNA 1xNR

NA: not applicable, NR: not reported.

For each item present, one point was awarded and added up to an overall score. The methodological quality of the studies was qualified as ‘good’ with a minimum of 10 points, ‘fair’ with a score of 8 or 9 points and ‘poor’ if 7 points or less were scored.

### 3.4 Study population

The majority of the included studies (74%) were conducted in Japan, except for 8 studies which were conducted in Italy [24,25], United Kingdom [16,26], Spain [26,27], Turkey [28], the Netherlands [18] and the United States [17].

The population of the majority of the studies (74%) consisted of community dwelling older adults. Three studies comprised both community dwelling and institutionalized older adults [22,26,29] and 3 studies included institutionalized older adults only [18,24,27]. Two studies included hospitalized patients [16,17] and one study comprised patients from a geriatric outpatient clinic and convalescent rehabilitation center [30].

Four studies compared septuagenarians and octogenarians [15,[31], [32], [33]]. Mean age of older adults in all other studies was above 85 years (4 studies), 80 (4 studies), 75 years (12 studies) and 70 years (9 studies). Only one study included a small subgroup aged 65–69 years [26]. In two studies only female older adults were included [20,22].

### 3.5 Masticatory function indicators

The majority of the studies (59%, n = 20) used one indicator for masticatory function, the other 41% used two (n = 9) [15,24,29,[34], [35], [36], [37], [38], [39]] or more (n = 5) [12,[21], [22], [23],^40^] indicators. The outcome of a combination of indicators was sometimes referred to as oral frailty [40] or oral or masticatory (hypo)function [15,22,23,34].

Most studies (79%, n = 27) used the maximum occlusal force (MOF). To assess the MOF, subjects were asked to bite on a horseshoe-shaped pressure-sensitive film which was digitally analyzed with a computer and resulted in a value for occlusal force in Newton. MOF was used as a single indicator in 11 studies [11,13,14,19,20,26,28,31,32,41,42]. The next most used test was the mixing ability test or gum jelly test (41%, n = 14). Mixing ability of a two-colored chewing gum was either visually or computer analyzed. Particularization of a gum jelly test was done visually or by measuring salivary glucose levels after chewing. It was used as a single indicator in 5 studies [18,25,28,30,43]. MOF and a mixing ability test were combined in 4 studies [23,35,36,44] and in another 2 studies with an additional questionnaire on chewing function [12,21]. In 4 studies MOF was combined with either number of (functional) teeth [15,22,34] or with a questionnaire on chewing function [22,37]. The mixing ability or gum jelly test was in 2 studies combined with number of teeth [40] or with a questionnaire on chewing function [24,40]. Two studies used ultrasonography (US) to assess muscle thickness of the musculus masseter [27] or the musculus temporalis [29] and two studies used CT images to evaluate musculus masseter area [16,17]. Two studies combined masseter thickness measured by US in combination with MOF [38,39]. One study used additionally masseter muscle echo intensity, representing the fat and fibrous tissue in muscle, which can be used for diagnosis of sarcopenia [39].

### 3.6 Identified health outcomes

The health outcomes that have been identified are clustered in six categories: physical parameters and sarcopenia (12 studies), history of falling (2 studies), nutritional status (9 studies), frailty (8 studies), cognitive function (8 studies) and mortality (4 studies). Seven studies analyzed health outcomes from two or more categories [20,24,27,28,39,41,43].

Different physical parameters and parameters for sarcopenia were used. All but two studies used handgrip strength [26,41,42], gait speed [31], or both [19,20], sometimes additionally combined with other measures, including muscle mass measurement by bioimpedance analysis or ultrasound imaging to identify sarcopenia [24,27,28,39,43]. One study used vertical jump height as the only outcome parameter [35].

Nutritional status was evaluated using different (combinations of) assessment methods; the mini nutritional assessment (-short form) MNA(-SF) [24,27,28,40], the ‘global leadership initiative on malnutrition (GLIM)’ criteria [27], serum albumin level [30,37,40], anthropometric measures (body mass index [BMI], waist circumference ([WC] [24,25], biceps circumference [BC] [24] or calf circumference [CC]) [28,39] and bio-electrical impedance analyses [24,25,39].

Frailty was assessed by either focusing on the physical aspect of frailty [14,43], whereas other studies used a multidimensional assessment that also included psychological and activities of daily living components (Kihon Checklist [12,21,23,43], Clinical Frailty scale [28] or frailty based on limitations in ≥3 of the domains mobility, strength, endurance, physical activity, and nutritional status [38]) One study used incidence of functional disability as a health outcome, based on the first certification of long-term care insurance in Japan [34]. This criterion reflects assistance in the daily activities of frail and elderly individuals and is used in several epidemiological studies. We categorized this criterium in the outcome ‘frailty’.

Cognitive function was assessed by the Mini-Mental State Examination (MMSE) in 4 studies [18,20,28,36], the Japanese version of the Montreal Cognitive Assessment (MoCA-J) in 3 studies [15,32,33], and the Revised Hasegawa Dementia Rating scale (HDS-R) in one study [22].

### 3.7 Associations between health outcomes and masticatory function (Tables 2A, 2B and 2C)

The associations between health outcomes and masticatory function of the prospective cohort studies are displayed in Table 2A, of the retrospective cohort studies in Table 2B and in the cross-sectional studies in Table 2C. Studies are categorized by identified health outcomes and arranged by type of statistical analysis (multivariate, multivariable and simple). When multivariable analyses were performed, only these results are displayed.

**Table 2A:** Prospective cohort studies.

Study (year, country)	Follow-up (years)	Cohort	Masticatory function assessment	Outcome	Analysis	Significant findings
Komiyama et al. [34] (2020, Japan)	8 (median)	Community dwelling older adults (≥70 years) 815 subjects mean 75.1 ± 4.5 years 48% male	Reduced oral function, i.e., - MOF < 200 N - Number of remaining teeth <20	**Frailty** (Incidence of functional disability based on the first certification of long-term care insurance)	Multivariate (Cox proportional hazard models)	MOF < 200 N was associated with increased risk of functional disability (adjusted HR: 1.33; 95% CI: 1.04−1.72, p = 0.025) Number of teeth < 20 was associated with increased risk of functional disability (adjusted HR: 1.40; 95% CI: 1.07−1.84, p = 0.014).
Iwasaki et al. [14] (2018, Japan)	5	Community-dwelling older adults (aged 75 years at baseline) 322 subjects 75 ± 1 years 46% male	MOF	**Frailty** incidence (phenotype model-assessed frailty status with CHS)	Multivariable (Cox proportional hazards regression model)	During follow-up, 15% (n = 49) developed frailty. Subjects with lower MOF had a greater risk of frailty (adjusted HR for frailty in the upper through lower tertiles of MOF: 2.78, 95% CI: 1.15−6.72, p = 0.02, p for trend p < 0.01).
Horibe et al. [12] (2018, Japan)	2	Community dwelling older adults (≥65 years) 418 subjects 73.5 ± 5.6 years 42% male	- MOF - Mixing ability test (analyzed with a color chart) - Subjective chewing ability (questionnaire)	**Frailty** progression (determined with the Kihon Checklist)	Multivariable (multivariable logistic regression analyses)	Mixing ability and subjective chewing ability were related to frailty progression (- = prefrail to frail), (OR:1.49, 95% CI: 1.14−1.96, p < 0.01 for mixing ability and OR 0.59, 95% CI 0.36−0.99, p = 0.04 for subjective chewing ability). MOF was not related to frailty progression (prefrail-frail), however it was related to frailty compared to robust groups (robust-frail) (OR: 2.02 95% CI: 1.04–3.91, p 0.04
Hatta et al. [15] (2020, Japan)	3	Community dwelling older adults, aged 70 (n = 423) and 80 (n = 437) years old 860 subjects 70 ± 1 years and 80 ± 1 years 47.4 % male	Oral function: i.e.,: - MOF - nr. of (functional) teeth	**Cognitive function** (MoCA-J)	Multivariate (generalized estimating equation)	Subjects with greater MOF and higher number of teeth were associated with better cognitive function at a follow up of 3 years (estimate 0.162, p < 0.01, SE 0.035 for MOF, estimate 0.041, p < 0.01, SE 0.012 for number of (functional) teeth).
Iinuma et al. [11] (2016, Japan)	3	Community dwelling older adults (≥85 years) 489 subjects 87.3 ± 2.2 years 44.7% male	MOF	**Mortality**	Multivariate (multivariate Cox proportional hazards regression model)	MOF tertiles (lowest as a reference) was independently associated with a 0.7-fold lower risk of 3-year survival, even with handgrip strength added as a confounder (adjusted HR: 0.73, 95 % CI = 0.54–0.99, p = 0.040).
Ohi et al. [13] (2020, Japan)	13	Community dwelling older adults 815 subjects 75.1 ± 4.5 years 48.5% male	MOF	**Mortality**	Multivariable (Cox proportional hazards model)	Subjects with lower MOF were at greater risk of all-cause mortality (lower tertile of MOF versus the upper tertile, adjusted HR: 1.62; 95% CI: 1.05–2.51, p = 0.029, p for trend = 0.028 for males, adjusted HR: 1.94; 95% CI: 1.10–3.56, p = 0.022, p for trend = 0.022 for females)

CHS: cardiovascular health study, CI: confidence interval, HR: hazards ratio, MoCA-J: Japanese version of the Montreal cognitive assessment, MOF: maximum occlusal force, OR: odds ratio, SE: standard error.

**Table 2B:** Retrospective cohort studies.

Study (year, country)	Observation period (years)	Demographics	Masticatory function assessment	Outcome	Analysis	Significant findings
Waduud et al. [16] (2020, United Kingdom)	3.5 (median)	Carotid artery stenosis patients who underwent carotid endarterectomy and CT imaging 149 subjects 71.5 ± 2.1 years 63.8% male	Masseter muscle quantity measured on head CT scan (total masseter area, standardized against cranium circumference) and quality [total masseter density])	**Mortality**	Multivariable (Cox proportional hazards regression analyses)	Standardized masseter muscle area was predictive of post-operative overall (30 days, 1 year, 4 years) all-cause mortality (adjusted HR: 0.38, 95% CI 0.15–0.97, p = 0.043).
Wallace et al. [17] (2017, United States)	2	Blunt-injured trauma patients (≥65 years) 487 subjects 80.0 ± 8.6 years 62.6% male	Masseter muscle cross-sectional area (measured on head CT scan)	**Mortality**	Multivariable (Cox proportional hazards model)	Declining masseter muscle area was associated with decreased 2-year survival (adjusted HR: 0.76; 95% CI 0.60–0.96, p = 0.023).

CI: confidence interval, CT: computed tomography, HR: hazards ratio.

**Table 2C:** Cross-sectional studies.

Study (year, country)	Demographics	Masticatory function assessment	Outcome: Physical parameters and sarcopenia	Analysis	Significant findings
Gonzalez-Fernandez et al. [27] (2021, Spain)	Institutionalized older adults 464 subjects mean 84.7 ± 7.7 years 30% male	Masseter MT by US	Sarcopenia (muscle strength [HGS], muscle quantity [bioimpedance measures] and physical performance [gait speed])	Multivariable (multiple logistic regression analysis, forced entry method)	Masseter MT is associated with sarcopenia: a 1 mm decrease in masseter MT increased the risk of sarcopenia by ∼57% (adjusted OR: 0.43, 95% CI: 0.34–0.54, p < 0.001)
Hihara et al. [19] (2020, Japan)	Community dwelling older adults 272 subjects mean 75.1 ± 7.5 years 37% male	MOF	- HGS - Gait speed	Multivariable (covariance structure analyses)	Positive correlations were found between HGS, gait speed and MOF scores for both sexes. (HGS: beta = 0.187, p < 0.05, gait speed: beta = 0.455, p < 0.01 for male; HGS: beta = 0.207, p < 0.01, gait speed: beta = 0.288, p < 0.01 for female)
Hirao et al. [20] (2014, Japan)	Community dwelling older adults 104 subjects Mean 74.6 ± 5.7 years All female	MOF	- HGS - Gait speed - lower extremity performance (strength of quadriceps, 30 seconds chair-stand test, maximal knee extensor strength, one-leg standing time with eyes open) - sit ups - length of forward bending - obstacle walking test	Multivariable (stepwise multiple regression analyses)	Only the 30 seconds chair-stand test was independently related to MOF (beta = 0.44, p < 0.01, R^2^ = 0.19), i.e., the higher the number in the 30 seconds chair-stand test, the greater MOF.
Morita et al. [35] (2018, Japan)	Community dwelling older adults (≥65 years) 231 subjects mean 74.4 ± 5.6 years) 23% male	- MOF - Gum jelly test (measured by salivary glucose concentration after chewing)	Vertical jump height	Multivariable (multiple regression analyses)	Multiple regression analyses with age as a covariate revealed vertical jump height to be predicted by masticatory performance by gum jelly test (p = 0.02) and MOF in female (p < 0.001).
Okada et al. [31] (2015, Japan)	Community dwelling older adults 655 septuagenarians Mean 70 ± 1 years 49% male 629 octogenarians Mean 80 ± 1 years 42% male	MOF	Gait speed	Multivariable (structural equation modeling analyses)	Gait speed was associated with MOF (standardized direct effect = 0.11, p < 0.001).
Ozsürekci et al. [28] (2022, Turkey)	Community dwelling older adults 135 subjects 75.7 ± 7.2 years 44% male	Mixing ability test (analyzed with a color chart)	Sarcopenia (assessed by muscle strength (HGS), muscle mass (bio-impedance analyses and gastrocnemius and masseter MT by US) and physical performance (gait speed)	Multivariable (multivariable linear regression analyses)	Only in females, low hand grip strength was associated with poor chewing (beta = −0.256, p < 0.05, R^2^ = 0.289).
Yamaguchi et al. [39] (2019, Japan)	Community dwelling older adults 139 subjects Mean 75 ± 4 years 47% male	- masseter muscle quality measured by echo intensity (MMEI) - masseter MT by US - MOF	- HGS - Gait speed	Multivariable (multiple regression analyses)	MMEI was positively correlated with masseter MT, HGS and gait speed (not with MOF). (beta = −0.31, p < 0.01, 95% CI; −2.16 to -0.65 for masseter MT, −0.32, p < 0.01, 95% CI − 0.84 to 0.16 for HGS and beta = −0.25, p < 0.01, 95% CI; −13.11 to -2.73 for gait speed, adjusted R^2^ = 0.30).
Aquilanti et al. [24] (2021, Italy)	Institutionalized older adults 32 subjects mean 86.7 ± 5.7 years 25% male	-Mixing ability test (computer analyzed) -Self-reported masticatory difficulties using a 0−10 VAS	Sarcopenia (muscle mass [bioimpedance analyses], muscle performance [gait speed test], muscle strength [HGS])	Simple (Pearson’s correlation)	81% (n = 26) were diagnosed with probable, confirmed or severe sarcopenia. No clear associations between impairment of masticatory function and the diagnosis of sarcopenia.
Eto et al. [41] (2018, Japan	Community dwelling older adults 159 subjects mean 74.3 ± 8.2 years 35% male	MOF	- HGS - Lower extremity performance (maximal knee extensor strength, one-leg standing time with eyes open)	Simple (Pearson’s correlation) Male and female analyzed together	MOF was positively correlated with HGS (Pearson’s correlation coefficient 0.382, p < 0.01) and lower extremity performance (maximal knee extensor strength and one-leg standing time, Pearson’s correlation coefficient 0.263 and 0.369, p < 0.01)
Laguna et al. [26] (2015, UK, Spain)	Institutionalized and community dwelling older adults (≥65 years) 103 subjects in the UK 27.2% male 100 subjects in Spain 38% male	MOF	HGS	Simple (Pearson’s Correlation) Male and female analyzed together	Positive correlation of HGS with MOF (Pearson’s correlation coefficient UK: 0.351; Spain: 0.427, p < 0.01).
Iinuma et al. [42] (2012, Japan)	Community dwelling older adults (≥85 years) 489 subjects 87.8 ± 2.2 years 44% male 87.3 ± 2.2 years 45% male	MOF	- HGS - lower extremity performance (timed timed up and go test, times chair standing test and one-leg standing test with eyes open)	Simple (Kruskal–Wallis test)	Higher levels of MOF were associated with better physical performance in men for all tests (handgrip strength p < 0.001, timed up and go test p < 0.003, timed chair standing test p < 0.01, one-leg standing time, p < 0.03). In women, the results did not reach statistical significance.
Yoshida et al. [43] (2022, Japan)	Community dwelling older adults 340 subjects mean 75.0 years 20% male	Salivary glucose concentration after chewing gum jelly	Sarcopenia (muscle mass [bioimpedance measures], muscle strength [HGS], physical performance [gait speed], according to AGWS)	Simple (Kruskal-Wallis Test, Mann-Whitney U test)	Lower masticatory function is associated with sarcopenia (p < 0.004)
**Study** **(year, country)**	**Demographics**	**Masticatory function assessment**	**Outcome: History of falling**	**Analysis**	**Significant findings**
Eto et al. [41] (2018, Japan)	Community dwelling older adults 159 subjects mean 74.3 ± 8.2 years 35% male	MOF	Falling in the past year	Simple (simple logistic regression analyses)	25% (n = 38) experienced falls in the past year. Falling experience was associated with a higher score of MOF (OR: 0.49; 95% CI: 0.09–2.62, p = 0.004).
Hasegawa et al. [44] (2019, Japan)	Community dwelling older adults 672 subjects mean 72.8 ± 5.9 years 33% male	- MOF - gum jelly test (particularization evaluated with a chart)	Falling in the past year	Simple (simple logistic regression analyses)	23% (n = 151) experienced falls in the past year. Falling experience was not associated with MOF or masticatory performance.
**Study** **(year, country)**	**Demographics**	**Masticatory function assessment**	**Outcome: Nutritional status**	**Analysis**	**Significant findings**
Okada et al. [30] (2010, Japan)	Older adult recruited from a geriatric outpatient clinic and convalescent rehabilitation hospital 200 subjects Mean 76.6 ± 7.1 years 39% male	Mixing ability test (computer analyzed)	Serum albumin level	Multivariable (Step-wise multiple linear regression)	Masticatory function is a predictor of serum albumin level (beta = 0.164, p = 0.045, adjusted R^2^ = 0.264).
Hasegawa et al. [29] (2019, Japan)	Older adults recruited from a long-term care hospital, a chronic care and rehabilitation unit of a general hospital, and a community walking group. 73 subjects Mean 81.3 ± 9.0 years 44% male	Temporal MT by US	BMI	Multivariable (multiple regression analyses)	Temporal MT was associated with BMI after adjusting for possible confounding factors of masticatory status, age and sex (beta = 0.335, p = 0.007).
Okamoto et al. [37] (2019, Japan)	Community dwelling older adults 3134 subjects 71.0 ± 4 years 51% male	- MOF - Questionnaire on chewable food	- Serum albumin levels - BMI	Multivariable (logistic regression analyses)	Only in females, low masticatory function (MOF < 100 N) was associated with low serum albumin levels (adjusted OR = 1.95; 95% CI = 1.15–3.31, p = 0.014) and low masticatory function (questionnaire) was associated with BMI (adjusted OR = 0.62; 95% CI = 0.46–0.85, p = 0.003).
Yamaguchi et al. [39] (2019, Japan)	Community dwelling older adults 139 subjects Mean 75 ± 4 years 46% male	Masseter muscle quality measured by echo intensity (MMEI)	- BMI, CC - Bioimpedance measurements	Multivariable (multiple regression analyses)	MMEI was negatively correlated with BMI, not with CC and bioimpedance measurements (beta = −0.21, 95% CI − 1.51 to -0.03, p < 0.05).
Iwasaki et al. [40] (2020, Japan)	Community dwelling older adults (≥70 years) 1054 subjects Mean 77 ± 4.8 years 41% male	Oral frailty defined as ≥ 3 items on: (1) <20 remaining teeth, (2) low masticatory performance (computerized analyses of increased surface area of comminuted gum jelly), (3) low articulatory oral motor skill, (4) low tongue pressure, difficulties in (5) eating and (6) swallowing (by questionnaire)	- MNA-SF - Serum albumin level	Multivariable (multivariable logistic regression analyses)	Oral frailty was observed in 20% of subjects (n = 217). Subjects with oral frailty had greater odds of more severe malnutrition (adjusted OR: 2.17; 95% CI: 1.58–2.98, p < 0.01) and serum albumin level (adjusted OR: 1.59; 95% CI: 1.10–2.31, p < 0.01).
Aquilanti et al. [25] (2020, Italy)	Community dwelling older adults 76 subjects mean 75.8 ± 5.6 years 55% male	Mixing ability test (computer analyzed)	- BMI, WC - Bioimpedance measurements	Simple	No association between reduced masticatory performance and the assessed parameters for nutritional status
Aquilanti et al. [24] (2021, Italy)	Institutionalized older adults 32 subjects mean 86.7 ± 5.7 years 25% male	Mixing ability test (computer analyzed)	- BMI, WC, BC - MNA - Bioimpedance measurements	Simple (Pearsons correlation coefficient)	No associations among masticatory performance and the studied nutritional and anthropometric parameters
Gonzalez-Fernandez et al. [27] (2021, Spain)	Institutionalized older adults 464 subjects mean 84.7 ± 7.7 years 30% male	Masseter MT by US	- GLIM criteria - MNA-SF - Meal forms assessment (diet of normal, soft or pureed texture)	Multivariable (logistic multivariable analyses)	Masseter MT is associated with malnutrition: a 1 mm decrease in masseter MT increased the risk of malnutrition by ∼63% (adjusted OR: 0.37, 95% CI: 0.29–0.49, p < 0.001) for MNA-SF or ∼34% (adjusted OR: 0.66, 95% CI 0.51–0.87, p < 0.003) for GLIM-criteria
Ozsürekci et al. [28] (2022, Turkey)	Community dwelling older adults 135 subjects 75.7 ± 7.2 years 44% male	Mixing ability test (analyzed with a color chart)	- BMI, CC - MNA-SF	Multivariable (multivariable linear regression analyses)	No association between reduced masticatory performance and BMI, CC, or MNA-SF
**Study** **(year, country)**	**Demographics**	**Masticatory function assessment**	**Outcome: Frailty**	**Analysis**	**Significant findings**
Watanabe et al. [38] (2017, Japan)	Community-dwelling older adults (≥65 years) 4720 subjects 72.1 ± 5.6 years 48% male	- MOF - Masseter MT by US	Frailty based on limitations in ≥3 of the domains mobility, strength, endurance, physical activity, and nutritional status	Multivariable (multivariable logistic regression analyses)	For each 100 N increase in MOF and 1 mm increase in masseter MT, the risk of frailty became significantly lower (OR: 0.94, 95% CI 0.90–0.99, p = 0.013 for MOF and OR: 0.89, 95% CI = 0.80–1.00, p = 0.040 for masseter MT).
Horibe et al. [21] (2018, Japan)	Community dwelling older adults (≥65 years) 659 subjects 72.7 ± 5.2 years 40% male	- MOF - Mixing ability test (analyzed with a color chart) - Subjective chewing ability (questionnaire)	Kihon Checklist	Multivariable (multivariable logistic regression analyses)	MOF, mixing ability and subjective chewing ability were related to frailty (robust vs (pre)frail group) (OR: 2.02, 95% CI: 1.04–3.91, p = 0.04 for MOF, OR: 1.66, 95% CI 1.02–2.70, p = 0.04 for mixing ability and OR: 5.61, 95% CI: 3.05–10.33, p < 0.01 for subjective mixing ability).
Shimazaki et al. [23] (2020, Japan)	Community-dwelling older adults (≥65 years) 973 subjects 73 ± 5 years 48% male	Oral hypofunction defined as ≥3 items on: - MOF - masticatory function (measured by salivary glucose concentration after chewing gum jelly - oral hygiene - oral dryness - longue-lip motor function - tongue pressure - swallowing function	Kihon Checklist	Multivariable (multinominal logistic regression analyses)	9.0% (n = 88) were frail. Approximately 60% of subjects had oral hypofunction. Subjects with oral hypofunction had higher ORs for frailty (adjusted OR: 2.0, 95% CI 1.2–3.5, p < 0.01) The risk for frail status was higher with reduced MOF (<500 N, adjusted OR: 2.2, 95% CI 1.3–3.8, p = 0.003), reduced tongue–lip motor function (adjusted OR 2.2, 95% CI 1.1–4.6, p = 0.04) and deteriorated swallowing (adjusted OR: 5.3, 95% CI 2.8–10.0, p < 0.001), not with masticatory function).
Yoshida et al. [43] (2022, Japan)	Community dwelling older adults 340 subjects Mean 75.0 years 20.3% male	Salivary glucose concentration after chewing gum jelly	- Kihon Checklist - phenotype model-assessed frailty status with CHS)	Multivariable (multivariable logistic regression analyses)	Lower masticatory function is associated with frailty determined with the Kihon Checklist (beta = 0.438, p = 0.17), not with the phenotype model-assessed frailty status with CHS)
Ozsürekci et al. [28] (2022, Turkey)	Community dwelling older adults 135 subjects 75.7 ± 7.2 years 43.7% male	Mixing ability test (analyzed with a color chart)	Clinical frailty scale	Simple (Mann‐Whitney U test)	20% (n = 27) had poor chewing function and had higher frailty scores (p = 0.017)
**Study** **(year, country)**	**Demographics**	**Masticatory function assessment**	**Outcome: Cognitive function**	**Analysis**	**Significant findings**
Ikebe et al. [33] (2018, Japan)	Community dwelling older adults aged 70 (n = 994) and 80 (n = 968) years old 994 subjects 70 ± 1 years 47.9% male 968 subjects 80 ± 1 years 47.0% male	MOF	MoCA-J	Multivariable (multiple linear regression analyses)	Higher MOF was correlated with better cognitive function (beta = 0.125, p < 0.001, adjusted R^2^ = 0.212)
Hirao et al. [20] (2014, Japan)	Community dwelling older female adults 104 subjects Mean 74.6 ± 5.7 years All female	MOF	MMSE	Multivariable (stepwise multiple regression analyses)	No association between MOF and cognitive function
Kugimiya et al. [36] (2019, Japan)	Community dwelling older adults (≥70 years) 1118 subjects 77.0 ± 4.7 years 39.8% male	- Gum jelly test (particularization analyzed with a chart) - MOF	MMSE	Multivariable (multiple linear regression analyses)	No association of MMSE with gum jelly test and MOF.
Takeshita et al. [32] (2016, Japan)	Community dwelling older adults aged 70 (n = 994) and 80 (n = 968) years old 39.9% male	MOF	MoCA-J	Multivariable (multiple linear regression analyses)	Higher MOF was correlated with better cognitive function (beta = 0.109, p = 0.017, adjusted R^2^ = 0.144)
Ozsürekci et al. [28] (2022, Turkey)	Community dwelling older adults 135 subjects 75.7 ± 7.2 years 43.7% male	- Mixing ability test (analyzed with a color chart)	MMSE	Multivariable (linear regression analyses)	Only in males, MMSE score was negatively associated with gum color scores (beta = 0.405, p < 0.01, R^2 =^ 0.525)
Weijenberg et al. [18] (2015, The Netherlands)	Institutionalized older adults with dementia 58 subjects 85.3 ± 5.4 years 19.0% male	Mixing ability test (computer analyzed)	Several cognitive tests including MMSE	Multivariable (hierarchical regression analyses)	No association between mixing ability and general cognition due to missing values
Miura et al. [22] (2003, Japan)	Institutionalized older female patients (cognitively impaired) and community dwelling older female (cognitively normal) ≥65 years) 44 subjects, cognitively impaired 81.1 ± 5.5 years All female 44 subjects, cognitively normal 82.3 ± 8.0 years All female	- Mastication score (questionnaire) - MOF - Occlusal contact area - Nr. of teeth	HDS-R	Simple (Mann–Whitney test)	All items of masticatory function were lower in cognitively impaired subjects (mastication score p = 0.008, MOF p = 0.001, occlusal contact area p = 0.001, nr. of teeth p = 0.042)

AGWS: Asian working group for sarcopenia consensus, BC: biceps circumference, Beta: standardized partial regression coefficient, BMI: body mass index, CC: calf circumference, CHS: cardiovascular health study, CI: confidence interval, CT: computed tomography, GLIM: global leadership initiative on malnutrition, HDS-R: revised Hasegawa dementia rating scale, HGS: hand grip strength, HR: hazards ratio, MMEI: Masseter muscle echo intensity, MMSE: mini mental state evaluation, MNA(-SF): mini nutritional assessment (-short form), MoCA-J: Japanese version of the Montreal cognitive assessment, MOF: maximum occlusal force, MT: muscle thickness, OR: odds ratio, SE: standard error, US: ultrasonographic examination, VAS: visual analogue scale, WC: waist circumference.

#### 3.7.1 Prospective cohort studies (Table 2A)

##### 3.7.1.1 Outcome: frailty

Three studies with a follow up of median 8, 5 and 2 years respectively investigated the association between masticatory function and frailty [12,14,34]. Reduced oral function, defined as MOF < 200 N and less than 20 remaining teeth, was associated with an increased risk of functional disability [34]. Horibe et al. [12] found that both mixing ability and subjective chewing ability were related to frailty progression, whereas MOF was not. MOF was however associated with frailty incidence in the other longitudinal study [14]. The first study used a multidimensional assessment of frailty, the second mainly focused on the physical aspect of frailty.

##### 3.7.1.2 Outcome: cognitive function

One prospective study had cognitive function as outcome and found that older adults with greater MOF and higher number of teeth were associated with better cognitive function at a follow up of 3 years [15].

##### 3.7.1.3 Outcome: mortality

Two prospective studies assessed the relation of MOF with all-cause mortality [11,13]. Both studies found with multivariate analyses that lower MOF was associated with at greater risk of all-cause mortality at 3 and 13 year respectively.

#### 3.7.2 Retrospective cohort studies (Table 2B)

The other 2 studies with mortality as an outcome had a retrospective design and assessed total masseter area measured on head CT scans, in relation to all-cause mortality [16,17]. These studies found that declining masseter muscle area was associated with decreased 2-year [17] and 4-year [16] survival.

#### 3.7.3 Cross-sectional studies (Table 2C)

Regarding the outcome categories physical parameters and sarcopenia, history of falling and nutritional status, only cross-sectional studies were available.

##### 3.7.3.1 Outcome: physical parameters and sarcopenia

Studies with multivariable analyses revealed positive correlations between MOF scores, handgrip strength and gait speed and MOF scores for both man and women [19,31]. Simple analyses showed that MOF was positively correlated with both handgrip strength [26,41] and lower extremity performance [41] too. In contrast, a study comprising only females revealed that lower extremity performance, but not hand grip strength or gait speed, was correlated to MOF [20].

Other studies found different results for men and women; Multivariable analyses showed that only in women, but not in men, lower extremity performance (measured by vertical jump height) was found to be predicted by masticatory function by mixing test and MOF [35], and low hand grip strength was associated with low gum color scores (low gum color scores classified as low masticatory function) [28]. Simple analysis showed that MOF was positively associated with handgrip strength and lower extremity performance, however only in man and not in women [42]. In one study gender was not evaluated in the analyses [26].

Sarcopenia was associated with masseter muscle thickness measured by US [27]. Masseter muscle echo intensity, which can be used for diagnosis of sarcopenia, was in a multivariable model positively correlated with masseter muscle thickness, handgrip strength and gait speed, however not with MOF [39]. The two studies using simple analyses and a mixing test, showed contradictive results: one study found that lower masticatory function was associated with sarcopenia [43], while the other study did not find an association between impairment of masticatory function and the diagnosis of sarcopenia [24].

##### 3.7.3.2 Outcome: history of falling

Two studies addressed the association between history of falling and masticatory function [41,44] with contradictive results. Eto et al. [41] found with simple analysis that the MOF average score among subjects with fall experience was higher than that of subjects without falls, whereas in the other study, with a more than 4 times bigger patient group, it was concluded that masticatory function, measured with both MOF and a mixing test, was not associated with history of falling [44].

##### 3.7.3.3 Outcome: nutritional status

Conflicting results are reported concerning the relation masticatory function and nutritional status. Multivariable analyses showed no association between masticatory function and BMI, CC, or MNA-SF in one study [28], while another study (with an almost 8 times bigger study population) showed that malnutrition assessed with MNA-SF or serum albumin levels was associated with oral frailty, defined as less than 20 remaining teeth and low masticatory function [40]. Also, malnutrition characterized by either GLIM or MNA criteria, was associated with decreased masseter muscle thickness [27]. In addition, masseter muscle quality, measured by echo-intensity, and temporal muscle thickness were associated with BMI [29,39].

Okada et al. [30] found that masticatory function was related to serum albumin levels (β = 0.164, p = 0.045). In addition, low masticatory function, assessed by both MOF and a questionnaire, were associated with low serum albumin levels and low BMI, however only in females [37]. The simple analyses of Aquilanti et al. [24,25] found no associations among masticatory function and the studied nutritional and anthropometric parameters (MNA, bioimpedance analyses, BMI, BC, WC). Multivariable analyses did not show an association of bioimpedance analyses, CC and masseter muscle quality either [39].

##### 3.7.3.4 Outcome: frailty

The 3 studies using MOF all found that the risk of frail status was higher with reduced MOF [21,23,38]. This also held for masseter muscle thickness [38]. Along MOF, Shimazaki [23] used combined measurements to define oral hypofunction, and whereas MOF was related to frailty, masticatory function measured with a mixing test was not. The other 2 cross-sectional studies using the mixing test only, did however find that reduced masticatory function was associated with higher frailty scores [28,43].

##### 3.7.3.5 Outcome: cognitive function

Multivariable analyses showed MOF to be associated with cognitive function in 2 studies [32,33], however, in the other 2 studies, no association was found [20,36]. Of the 3 studies using the mixing test, multivariable analyses showed that only in males, MMSE score was negatively associated with gum color scores [28], the other 2 studies found no association [18,36]. Lastly, Miura et al. [22] found with simple analyses that subjective masticatory function, MOF, occlusal contact area and number of teeth were all lower in cognitively impaired subjects.

## 4 Discussion

The aim of this review was to assess if reduced masticatory function in older adults is related to adverse health outcomes. From this review, it can be concluded that the predictive value of masticatory function for all the adverse health outcomes (i.e., sarcopenia, history of falling, nutritional status, frailty, cognitive function and mortality) seems to be limited as the observed associations were generally low to moderate. These low to moderate associations can be due to the complex and multidimensional character of masticatory function and the multifactorial character of most adverse health outcomes.

All prospective studies included in this review found a relation between masticatory function and the assessed adverse health outcomes [11,13,34]. Prospective studies with ‘hard’ outcome measures as all-cause mortality or incident functional disability, based on the first certification of long-term care insurance, showed that a greater risk of these outcomes were associated with lower maximum occlusal force (MOF) or reduced oral function, defined as MOF less than 200 N and less than 20 remaining teeth. In addition, prospective studies showed that older adults with lower MOF had a greater risk of frailty [14], and that lower MOF and lower number of remaining teeth [15] were associated with cognitive decline. In addition, mixing ability and subjective chewing ability were related to frailty progression [12]. So, it can be concluded that reduced masticatory function is related to increased risk of frailty incidence and frailty progression, cognitive decline, and mortality. Regarding other physical measures and sarcopenia, history of falling and nutritional status, only cross sectional studies were available and results were less concordant. Although multivariable analyses were applied in the majority of the included studies, adjusting for all potential confounding factors is complex regarding the multidimensional character of masticatory function and multifactorial character of most adverse health outcomes.

A major limitation of this systematic review is the absence of a clear conceptual definition of masticatory function, a complex and multidimensional process. This implicates a lack of construct validation of the sometimes one-dimensional objective assessment methodologies.

The MOF was most frequently used objective assessment method. MOF is measured using a digital device and is easily performed and therefore applicable in older adults. In order to interpret MOF values, Komiyama et al. [34] set a threshold of 200 N, based on a previous study by Ikebe et al. [45]. However, MOF between men and women differs significantly [46] and distinction on gender is necessary. Indeed, gender related differences were reported regarding masticatory function and physical health outcomes as handgrip strength and lower extremity performance, however sometimes conflicting [28,35,42]. Also, Watanabe et al. [38] found that MOF in frail versus robust older adults differs in age groups. Since clear gender and age specific cut-off points for MOF are lacking, further research on valid cut-off points is needed to identify older adults at risk for adverse health outcomes. A limitation of MOF is that it focuses on the maximum generated force rather than functional chewing function. As functional chewing function is more related to coordinative function of masticatory muscles [47] including the tongue, and therefore associated with mixing function [48], it might be of greater relevance to assess functional chewing ability, despite the challenging implementation in older adults [18]. Subjective chewing function was also assessed, however, there is currently no patient-reported outcome measure for masticatory function in adults with high-level evidence for psychometric properties [49].

Another objective assessment method is imaging of masticatory muscles by ultrasonography or CT. Like MOF, measurement of masticatory muscle thickness is a one-dimensional approach for a complex and multidimensional process, and it can be argued whether this is a valid measurement for masticatory function. However, the dynamic change in the masseter muscle thickness between contraction and relaxation has been shown to be positively correlated with masticatory function in older adults [50]. It must be noted that thickness of masticatory muscles can be related to sarcopenia as well [27,39] and two of the six studies using imaging techniques of masticatory muscles it was effectively used as a marker for sarcopenia. Therefore, ultrasonography has a high potential in daily practice, since the masseter muscle is easily accessible and relatively cheap mobile devices available [51,52]. Assessment of the masseter muscle in older adults has been shown to be useful in prediction of short- and long-term postoperative complications [53].

Perhaps because of the absence of a clear conceptual definition for masticatory function, more than 40% of the included studies used more than one method to assess masticatory function. It seems to be worthful to assess masticatory function in a broader context of oral health or oral function. The importance of a multidimensional approach for both masticatory function and adverse health outcomes is illustrated by Iwasaki et al. [40], who performed a multifaceted oral health assessment, including number of remaining teeth, masticatory performance by mixing test, tongue pressure, and self-perceived difficulties in eating and swallowing. With this multidimensional approach for defining ‘oral frailty’, a relatively high odds ratio was reported for the relation with malnutrition assessed with MNA-SF (adjusted OR 2.17), also a multidimensional assessment. The reported odds ratio of ‘oral frailty’ with serum albumin level, a one-dimensional indirect measure of malnutrition, was lower in this study (adjusted OR 1.59). The importance of a multidimensional approach is also illustrated in the study of Shimazaki [23], who reported on 'oral hypofunction', including assessment of MOF, mixing test, tongue pressure and self-perceived swallowing function. Subjects with ‘oral hypofunction’ had adjusted an OR 2.0 for frailty, determined with the multidimensional Kihon Checklist, whereas masticatory function by mixing test alone was not associated with frailty.

An important finding was that decreased masseter muscle thickness increased the risk of malnutrition [27]. This highlights the interplay between sarcopenia, malnutrition and (oral) frailty, and shows the potency of masticatory muscle measurements in older adults. From a pathophysiological point of view, both malnutrition and sarcopenia share many components [54]. A significant decline in the intake of multiple nutrients (protein, sodium, potassium, calcium, vitamin A, vitamin E and dietary fibre) and food groups (vegetable and meat) has been shown in older adults with impaired dentition [55]. Thus, prevention of decline of oral function may lower the burden of malnutrition and sarcopenia for older adults. Moreover, MOF is correlated with cognitive function directly as well as indirectly via food intake [33]. Another possible mechanism that might explain the association between masticatory function and cognitive function is that muscle contraction during chewing increases middle cerebral arterial blood flow velocity, suggesting the relationship between cognition and masticatory function is based on blood flow [56].

The importance of maintaining masticatory function in relation to cognitive function is in line with the results of the meta-analysis of Cerruti-Kopplin [57], comprising 10 prospective cohort studies; Individuals with less than 20 teeth were at a 20% higher risk for developing cognitive impairment compared to those with more than 20 teeth. In addition, they found that unsatisfactory masticatory function was more frequent in edentulous older adults with low quality dentures compared with high quality dentures and corresponded with decreased cognitive function [58]. This demonstrates the importance of assessing denture use or -quality as a covariate. In this light, it must be noted that no or little information was given on oral health status or quality of dentures in the included studies of this review.

Lahoud et al. [59] identified in their recent review frailty, sarcopenia, cognitive decline and malnutrition as notable risk factors for masticatory dysfunction. The importance of maintenance of masticatory function in older adults who suffer from certain systemic diseases or physical dysfunction, and the need to improve their masticatory function to achieve healthy aging, has formerly been appointed by Yanpin Fan et al. [60]. They reported significant negative associations between masticatory function and stroke, sarcopenia, amyotrophic lateral sclerosis, chronic obstructive pulmonary disease, dyspepsia, dysphagia, anorexia, and carotid atherosclerosis. Dibello et al. [61] highlighted the importance of a multidimensional assessment of oral function and used the concept ‘oral frailty’ too; few remaining teeth, masticatory function, occlusal force and chewing difficulties were all considered to be associated with frailty.

The majority of the included study populations consisted of Japanese older adults, which can be regarded as another limitation of this systematic review; the results may not be representative for other populations or countries. Furthermore, because of a variety of methods identified for objectivating masticatory function and health outcomes, a meta-analyses could not be performed.

With clinical perspective in mind, this review emphasizes the importance of maintaining masticatory function in older adults. Previous studies showed improved masticatory function, measured by MOF and a mixing test, and increased masseter muscle thickness and -activity after prosthetic rehabilitation with (partial) implant-supported prosthesis [62,63], suggesting an important role for continuous dental treatment. Assessment of masticatory function, or oral function in a broader perspective, is relatively simple and could help to identify older adults at risk for adverse health outcomes. Ideally, these older adults are recognized early and receive adequate care to prevent further deterioration. Strategies for preventing tooth loss and adequate prosthodontic treatment during lifetime could maintain masticatory function in older adults, thereby possibly preventing adverse health outcomes and promoting healthy ageing.

## 5 Conclusion

Reduced masticatory function in older adults is associated with increased incidence of frailty, frailty progression, cognitive decline and mortality. Prevention of decline of masticatory function by adequate oral care may therefore contribute to healthy ageing.

## Author contributions

MDS, WN and AV conceived and designed the study. MDS and WN conducted the systematic search, screened articles, and read the full texts for eligibility. MDS and WN extracted data from the original studies and evaluated the studies for risk of bias. MDS, WN and MB contributed to the interpretation of the results and wrote the first draft of the manuscript. AV contributed to the interpretation of the results and critically revised the manuscript. All authors have read and approved the final manuscript. The corresponding author attests that all listed authors meet authorship criteria and that no others meeting the criteria have been omitted. The author’s report no conflict of interest.

## Funding

This study was funded by the author’s institutions.

## Conflict of interest

The authors declare that they have no competing interest.
