# Supplementary material for: The predictive value of masticatory function for adverse health outcomes in older adults: a systematic review
Source: J Nutr Health Aging. 2024 Mar 14;28(5):100210. doi: 10.1016/j.jnha.2024.100210 (PMC12877280; doi:10.1016/j.jnha.2024.100210)
Supplement: Supplementary file 1 [file mmc1.docx]

## Appendix: Search strategy

PI(C)O:

“What is, for older adults (P), in relation to their masticatory (muscle) function (I), the predictive value for (adverse) health outcomes (O)?”

**PubMed search**

("Aged"[Mesh] OR “aged”[tiab] OR “adult*”[tiab] OR “elder*”[tiab] OR “middle age*”[tiab] OR “older person*”[tiab] OR “older people*”[tiab] OR “older individual*”[tiab] OR “older subject*”[tiab] OR “older age”[tiab] OR “older aged”[tiab] OR “older patient*”[tiab] OR “older population*”[tiab] OR “senior*”[tiab] OR “very old”[tiab])

**AND**

("Masticatory Muscles"[Mesh] OR “masticatory muscle*”[tiab] OR “masseter muscle*”[tiab] OR “temporal muscle*”[tiab] OR "Bite Force"[Mesh] OR “bite force*”[tiab] OR “masticatory performance*”[tiab] OR “occlusal force*”[tiab])

**AND**

("Association"[Mesh] OR “associat*”[tiab] OR "Validation Studies as Topic"[Mesh] OR “validation stud*”[tiab] OR “validity of results”[tiab] OR “reliabilit*”[tiab] OR “prognos*”[tiab] OR “predict*”[tiab] OR “relationship*”[tiab])

**AND**

("Physical Fitness"[Mesh] OR “physical fitness*“[tiab] OR ”cardiorespiratory fitness”[tiab] OR “physical functional performance”[tiab] OR “gait analy*”[tiab] OR “gait speed*”[tiab] OR “physical status*“[tiab] OR "Mortality"[Mesh] OR “mortalit*“[tiab] OR “death rate*”[tiab] OR "Health Status"[Mesh] OR “health status*“[tiab] OR “health level*”[tiab] OR “geriatric assessment”[tiab] OR "Frailty"[Mesh] OR “frail*“[tiab] OR “debilit*”[tiab] OR "Atrophy"[Mesh] OR “atroph*”[tiab] OR “nutritional status*”[tiab] OR “sarcopenia”[tiab] OR “muscle mass*“[tiab] OR "Outcome Assessment, Health Care"[Mesh] OR (“health care*”[tiab] *AND* (“assessment*”[tiab] *OR* “outcome*”[tiab])) OR (“outcome”[tiab] *AND* (“stud*”[tiab] *OR* “assessment*”[tiab])) OR “functional assessment*“[tiab] OR “outcome measure*”[tiab] OR "Muscle Strength"[Mesh] OR “strength*”[tiab] OR “muscle inhibition*”[tiab] OR “handgrip*”[tiab] OR "Mental Processes"[Mesh] OR “mental proces*”[tiab] OR “human information processing*”[tiab] OR "Dementia"[MeSH] OR “dementia*”[tiab] OR “cognit*“[tiab] OR “alzheimer*“[tiab] OR “amenti*“[tiab] OR "Prognosis"[Mesh] OR “prognos*”[tiab] OR “general health“[tiab] OR “fall*“[tiab] OR “chewing problem*”[tiab] OR “malnutrition*”[tiab])

**NOT**

("Animals"[Mesh] NOT "Humans"[Mesh])

**Embase search**

(‘aged’/exp OR ‘aged’:ab,ti OR ‘adult*’:ab,ti OR ‘elder*’:ab,ti OR ‘middle age*’:ab,ti OR ‘older person*’:ab,ti OR ‘older people*’:ab,ti OR ‘older individual*’:ab,ti OR ‘older subject*’:ab,ti OR ‘older age’:ab,ti OR ‘older aged’:ab,ti OR ‘older patient*’:ab,ti OR ‘older population*’:ab,ti OR ‘senior*’:ab,ti OR ‘very old’:ab,ti)

**AND**

(‘masticatory muscles’/exp OR ‘masticatory muscle*’:ab,ti OR ‘masseter muscle*’:ab,ti OR ‘temporal muscle*’:ab,ti OR ‘occlusal force’/exp OR ‘bite force*’:ab,ti OR ‘masticatory performance*’:ab,ti OR ‘occlusal force*’:ab,ti)

**AND**

(‘association’/exp OR ‘associat*’:ab,ti OR ‘validation study’/exp OR ‘validation stud*’:ab,ti OR ‘validity of results’:ab,ti OR ‘reliabilit*’:ab,ti OR ‘prognos*’:ab,ti OR ‘predict*’:ab,ti OR ‘relationship*’:ab,ti)

**AND**

(‘fitness’/exp OR ‘fitness*’:ab,ti OR ‘physical functional performance*’:ab,ti OR ‘gait analy*’:ab,ti OR ‘gait speed*’:ab,ti OR ‘physical status’:ab,ti OR ‘strength*’:ab,ti OR ‘mortality’/exp OR ‘mortalit*’:ab,ti OR ‘death rate’:ab,ti OR ‘health status’/exp OR ‘health status’:ab,ti OR ‘health level*’:ti,ab OR ‘geriatric assessment’/exp OR ‘geriatric assessment’:ab,ti OR ‘frailty’/exp OR ‘frail*’:ab,ti OR ‘debilit*’ OR ‘muscle atrophy’/exp OR ‘muscle atrophy’:ab,ti ‘nutritional status’:ab,ti OR ‘sarcopenia’:ab,ti OR ‘muscle mass*’:ab,ti OR ‘outcome assessment’/exp OR ‘outcome assessment’:ab,ti OR (‘health care*’:ab,ti AND (‘assessment*’:ab,ti OR ‘outcome*’:ab,ti)) OR (‘outcome’:ab,ti AND (‘stud*’:ab,ti OR ‘assessment*’:ab,ti)) OR ‘functional assessment’:ab,ti OR ‘outcome measure*’:ab,ti OR ‘muscle strength’/exp OR ‘strength’:ab,ti OR ‘muscle inhibition’:ab,ti OR ‘handgrip*’:ab,ti OR ‘mental function’/exp OR ‘mental function’:ab,ti OR ‘dementia’/exp OR ‘dementia*’:ti,ab OR ‘cognit*’:ab,ti OR ‘alzheimer*’:ab,ti OR ‘amenti*’:ab,ti OR ‘prognosis’/exp OR ‘prognos*’:ab,ti OR ‘general health’:ab,ti OR ‘fall*’:ab,ti OR ‘chewing problem’:ab,ti OR ‘malnutrition’:ab,ti)

**Cinahl search**

(MH "Aged+" OR AB aged OR AB adult* OR AB elder* OR AB “middle age*” OR AB “older person*” OR AB “older people*” OR AB “older individual*” OR AB “older subject*” OR AB “older age” OR AB “older aged” OR AB “older patient*” OR “older population*” OR “senior*” OR “very old” OR TI (aged OR adult* OR elder* OR “middle age*” OR “older person*” OR “older people*” OR “older individual*” OR “older subject*” OR “older age” OR “older aged” OR “older patient*” OR “older population*” OR “senior*” OR “very old”))

**AND**

(MH "Masticatory Muscles+" OR AB (“masticatory muscle” OR AB “masseter muscle*” OR AB “temporal muscle*”) OR TI (“masticatory muscle” OR “masseter muscle*” OR “temporal muscle*”) OR MH "Bite Force" OR AB “bite force*” OR AB “masticatory performance*” OR AB “occlusal force*” OR TI (“bite force*” OR “masticatory performance*” OR “occlusal force*”))

**AND**

(AB associat* OR MH "Validation Studies+" OR AB “validation stud*” OR AB “validity of results” OR AB “reliabilit*” OR AB “predict*” OR AB “relationship*” OR TI (associate* OR “validation stud*” OR “validity of results” OR “reliabilit*” OR “predict*” OR “relationship*”))

**AND**

(MH "Physical Fitness+" OR AB “physical fitness*“ OR AB “cardiorespiratory fitness*“ OR AB “physical functional performance*“ OR AB “gait analy*“ OR AB “gait speed*“ OR AB “physical status*“ OR MH "Mortality+" OR AB “mortalit*“ OR AB “death rate*“ OR MH "Health Status+" OR AB “health status*“ OR AB “health level*“ OR AB “geriatric assessment*“ OR MH "Frailty Syndrome+" OR AB “frail*“ OR AB “debilit*“ OR MH "Atrophy+" OR AB “atroph*" OR AB “nutritional status*“ OR AB “sarcopenia“ OR AB “muscle mass*“ OR MH "Outcome Assessment+" OR (AB “health care*“ AND (AB “assessment*“ OR AB “outcome*“)) OR (AB “outcome“ AND (AB “stud*“ OR AB “assessment*“)) OR AB “functional assessment*“ OR AB “outcome measure*“ OR MH "Muscle Strength+" OR AB “strength*“ OR AB “muscle inhibition*“ OR AB “handgrip*“ OR MH "Mental Processes+" OR AB “mental process*“ OR AB “human information processing*“ OR MH "Dementia+" OR AB “dementia*“ OR AB “cognitive“ OR AB “alzheimer“ OR AB “amenti*“ OR MH "Prognosis+" OR AB “prognos*“ OR AB “general health“ OR AB “fall*“ OR AB “chewing problem*“ OR AB “malnutrition*“ OR TI “physical fitness*“ OR TI “cardiorespiratory fitness*“ OR TI “physical functional performance*“ OR TI “gait analy*“ OR TI “gait speed*“ OR TI “physical status*“ OR TI “mortalit*“ OR TI “death rate*“ OR TI “health status*“ OR TI “health level*“ OR TI “geriatric assessment*“ OR TI “frail*“ OR TI “debilit*“ OR TI “atroph*" OR TI “nutritional status*“ OR TI “sarcopenia“ OR TI “muscle mass*“ OR (TI “health care*“ AND (TI “assessment*“ OR TI “outcome*“)) OR (TI “outcome“ AND (TI “stud*“ OR TI “assessment*“)) OR TI “functional assessment*“ OR TI “outcome measure*“ OR TI “strength*“ OR TI “muscle inhibition*“ OR TI “handgrip*“ OR TI “mental process*“ OR TI “human information processing*“ OR TI “dementia*“ OR TI “cognitive“ OR TI “alzheimer“ OR TI “amenti*“ OR TI “prognos*“ OR TI “general health“ OR TI “fall*“ OR TI “chewing problem*“ OR TI “malnutrition*“)
